# Supplementary material for: Adsorption separation of heavier isotope gases in subnanometer carbon pores
Source: Nat Commun. 2021 Jan 22;12:546. doi: 10.1038/s41467-020-20744-6 (PMC7822881; doi:10.1038/s41467-020-20744-6)
Supplement: Supplementary file 1 — Supplementary Information [file 41467_2020_20744_MOESM1_ESM.pdf]

# Supplementary Information

## Adsorption Separation of Heavier Gases Isotope in Subnanometer Carbon Pores

Sanjeev Kumar Ujjain[1], Abhishek Bagusetty[2], Yuki Matsuda[3], Hideki Tanaka[1], Preety Ahuja[1], Carla de Tomas[4], Motomu Sakai[5], Fernando Vallejos-Burgos[1,6], Ryusuke Futamura[1], Irene Suarez-Martinez[4], Masahiko Matsukata[7], Akio Kodama[3], Giovanni Garberoglio[8,9], Yury Gogotsi[1,10], J. Karl Johnson[2], Katsumi Kaneko[1*]

*[1]Research Initiative for Supra-Materials, Shinshu University, Nagano-City, Japan*
*[2]Department of Chemical & Petroleum Engineering, University of Pittsburgh, Pennsylvania, USA*
*[3]Institute of Science and Engineering, Kanazawa University, Kanazawa, Japan*
*[4]Department of Physics and Astronomy, Curtin University, Perth, Australia*
*[5]Research Organization for Nano and Life Innovation, Waseda University, Tokyo, Japan*
*[6]Morgan Advanced Materials, Carbon Science Centre of Excellence, State College, PA 16803, USA*
*[7]School of Advanced Science and Engineering, Waseda University, Tokyo, Japan*
*[8]European Centre for Theoretical Studies in Nuclear Physics and Related Areas (FBK-ECT*), Strada delle Tabarelle 286, I-38123 Trento, Italy*
*[9]Trento Institute for Fundamental Physics and Applications (TIFPA-INFN), via Sommarive 18, I-38213 Trento, Italy*
*[10]Department of Material Science and Engineering, and A.J. Drexel Nanomaterials Institute, Drexel University, Pennsylvania, USA*

*Correspondence to: kkaneko@shinshu-u.ac.jp

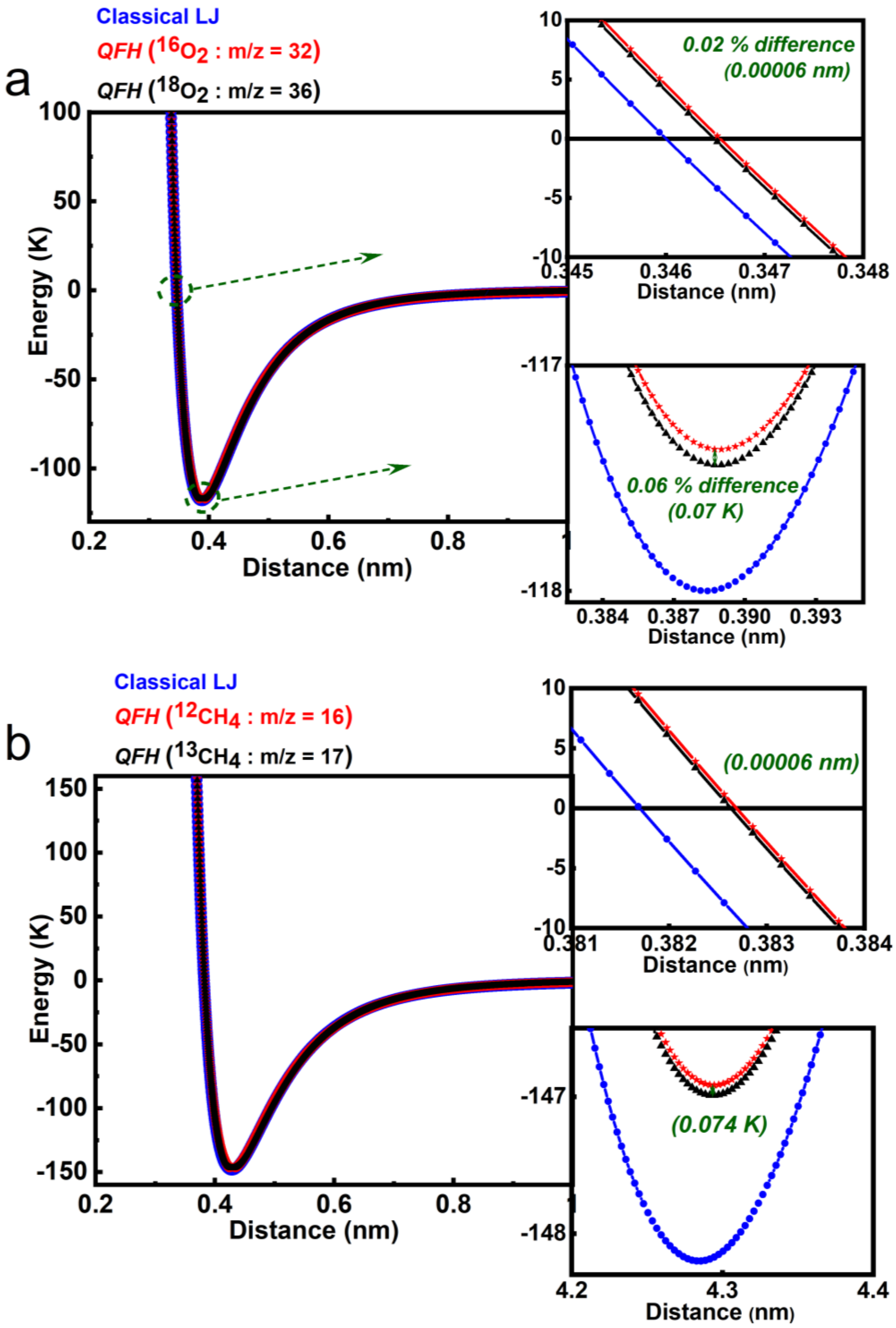


**Supplementary Figure 1. Interaction Potentials. a,** $O_2$-$O_2$ interaction potential: Lennard-Jones (LJ) potential (blue) and Quadratic Feynman-Hibbs effective potential (red: $^{16}O_2$, black: $^{18}O_2$). **b,** $CH_4$-$CH_4$ interaction potential: Lennard-Jones (LJ) potential (blue) and Quadratic Feynman-Hibbs effective potential (red: $^{12}CH_4$, black: $^{13}CH_4$).

**Supplementary Note 1:**

We performed quantum effective potential calculations using the de Broglie wavelength and interaction potential using the classical Lennard-Jones (LJ) and the quantum Feynman-Hibbs (QFH)[1] potentials at 112 K. The de Broglie wavelengths of $^{16}O_2$ and $^{18}O_2$ at 112 K are 0.0292 nm and 0.0275 nm, respectively, which results in a difference of 0.0017 nm. In addition, the de Broglie wavelengths of $^{12}CH_4$ and $^{13}CH_4$ at 112 K are 0.0412 nm and 0.0400 nm, respectively, which results in a difference of 0.0012 nm. The QFH effective potential calculation gives a very small difference of 0.07 K in the potential energy minimum for both $^{18}O_2$ / $^{16}O_2$ and $^{12}CH_4$ / $^{13}CH_4$.

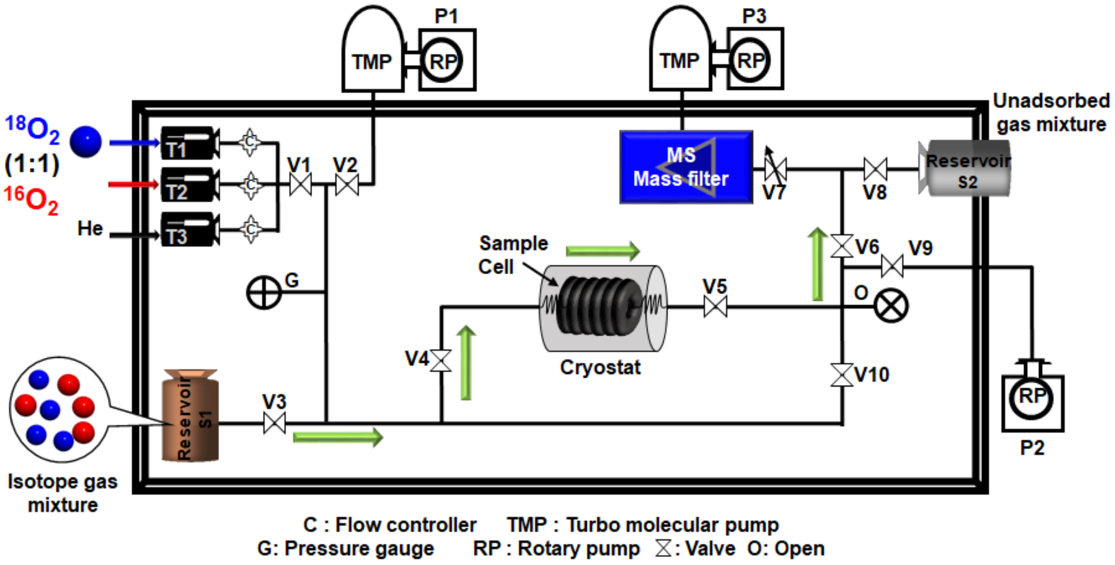


**Supplementary Figure 2. Working Instrument.** Schematic representation of the laboratory designed flow-type dynamic adsorption separation instrument.

## Supplementary Note 2:

The dynamic adsorption separation experiments were performed using in-lab assembled instrument schematically represented above. The instrument has been equipped with a Swagelok adsorption sample cell (SS-4-WVCR-6-DF; 316 Stainless Steel Welded VCR Face Seal Fitting with porous metal gasket) with an internal diameter of, 1/4 in. and length 2 in., which was packed with 50 mg of samples. In the experimental setup, the adsorption cell was installed in cryostat unit which allows for in-situ pre-treatment of the adsorbents under vacuum before the adsorption separation experiment performed at 112 K. The isotope mixed gas of $^{16}O_2$ and $^{18}O_2$ of the composition 1:1 was produced using flow controllers (C) and stored in reservoir tank (S1). The vacuum pumps P1 and P3 are used for evacuation while P2

is used during partial pressure analysis. Before each set of experiments, the mixed gas concentration was analyzed on-line with a quadrupole mass spectrometer (MS), the signal of which has been calibrated in advance as shown in Supplementary Figure 3.

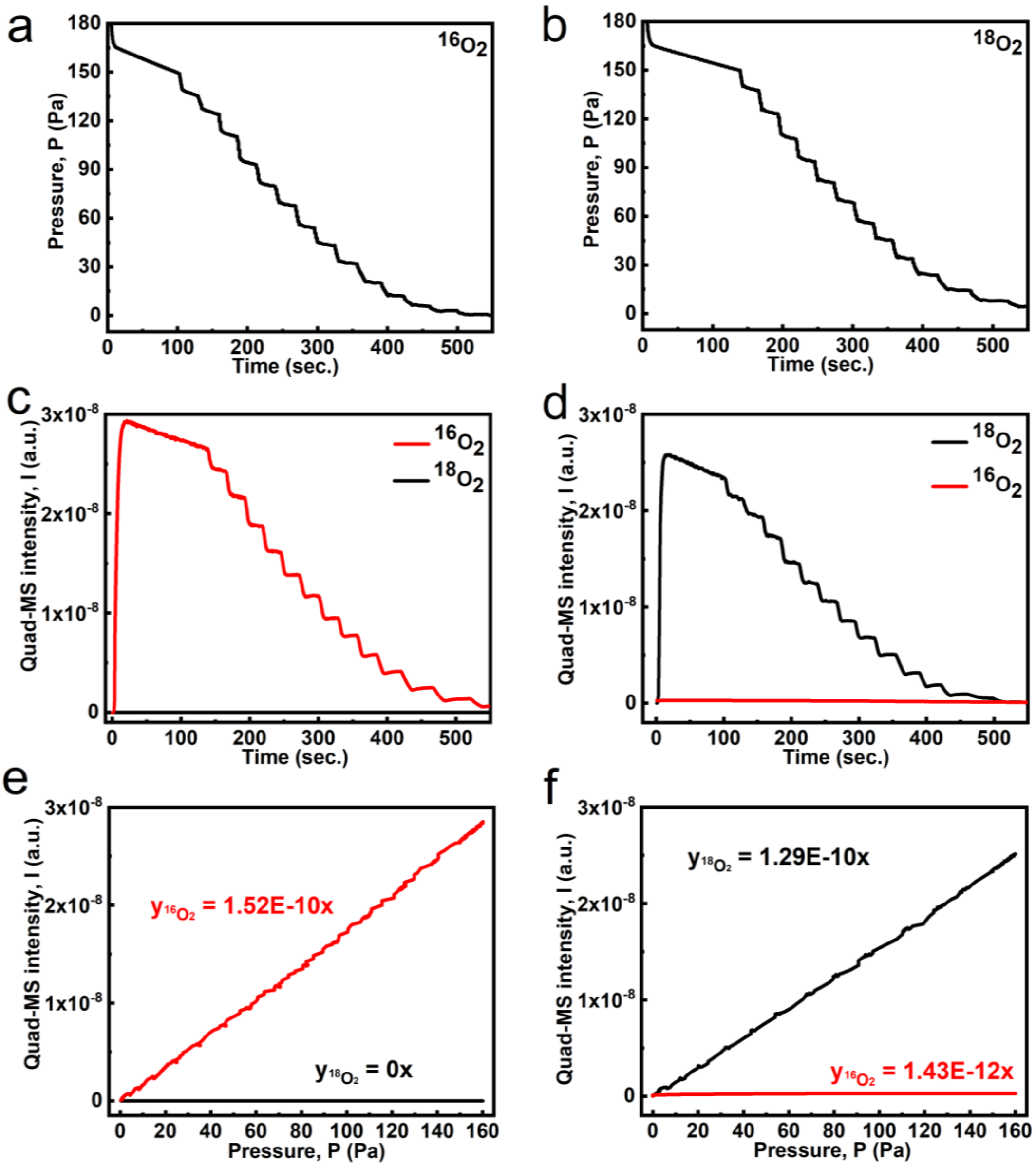


**Supplementary Figure 3. Correlation Curves. a & b,** Time course of partial pressure for $^{16}O_2$ and $^{18}O_2$. **c & d**, Time course of mass intensity for $^{16}O_2$ and $^{18}O_2$. **e & f,** Correlation of mass intensity versus partial pressure for $^{16}O_2$ and $^{18}O_2$, respectively.

**Supplementary Note 3:**

Using the relationship between linearity of "partial pressure" and "mass intensity" via calibration approaches, mole fractions of $^{18}O_2$ and $^{16}O_2$ in adsorbed phase and gas phase can be determined. Direct calibration for overlapping signals is given by:

$$I_i = K \sum a_{ij} \mathrm{P}_j$$

Where, $I_i$ - ion current of the mass M

K – instrument constant, related to the setup settings

$a_{ij}$ – calibration factor determined from the slope of partial pressure vs intensity

$P_j$ – partial pressure of the j-th component

For two component system, the linear equations could be written as follows:

$$\begin{bmatrix} I_1 \\ I_2 \end{bmatrix} = \begin{bmatrix} a_{11} & a_{21} \\ a_{12} & a_{22} \end{bmatrix} \cdot \begin{bmatrix} P_1 \\ P_2 \end{bmatrix}$$

i.e.

$$\begin{bmatrix} I_{36} \\ I_{32} \end{bmatrix} = \begin{bmatrix} a_{18O2(36)} & a_{16O2(36)} \\ a_{18O2(32)} & a_{16O2(32)} \end{bmatrix} \cdot \begin{bmatrix} P_{36} \\ P_{32} \end{bmatrix}$$

Such linear equations can be solved considering the following example:

$$X = ak + bq$$

$$Y = ar + bs$$

then,

$$\begin{bmatrix} k & q \\ r & s \end{bmatrix} \cdot \begin{bmatrix} a \\ b \end{bmatrix} = \begin{bmatrix} X \\ Y \end{bmatrix}$$

[Calibration factor] . [Unknown concentration] = [Experimental ion currents]

i.e.

$$\begin{bmatrix} a \\ b \end{bmatrix} = \begin{bmatrix} k & q \\ r & s \end{bmatrix}^{-1} \cdot \begin{bmatrix} X \\ Y \end{bmatrix}$$

this implies $a = \frac{sx-qy}{ks-qr}$ $\quad b = \frac{ky-rx}{ks-qr}$ $\quad {}^{b}/_{a} = \frac{ky-rx}{sx-qy}$

This is how the amount or mole fraction of $^{18}O_2$ and $^{16}O_2$ can be determined separately from a mixture in adsorbed phase and gas phase. The background contributions towards mass intensity form $^{18}O_2$ and $^{16}O_2$ should be subtracted before the mole fraction determination. During calibration, the needle valve V7 plays very significant role to avoid the deterioration of MS filament. The partial pressure analyzers are calibrated using higher pressures. Under these conditions, in order to safeguard the filament from deterioration, it is highly desirable to decrease the electron current to a very low level. In addition, bombarded surfaces by the non-transmitted ions which at many instances results in insulating layers receive less abuse. Furthermore, along with the possible performance deterioration of the instrument because of operating at high pressures, the greater density of molecules also interferes with the operation of the MS because of ion-molecule collisions. So, the needle valve V7 is opened to sufficiently large extent which protects the MS filter, while not obstructing the flow of mixed gas, as that is always less compared to maximum allowed value.

The flow rate of the mixed isotope feed gases 120 Pa ($^{12}CH_4$ + $^{13}CH_4$) or ($^{16}O_2$ + $^{18}O_2$) having different concentration of $^{18}O_2$ (4.8 at.% to 70 at.%) through the adsorption sample cell was regulated by mass flow controllers (~1 ml min$^{-1}$). The mixed $O_2$ gas having $^{16}O_2$ : $^{18}O_2$ = 1:1 is termed as equimolar feed gas. The composition changes of the mixed gas stream at the outlet was analyzed with the MS. Before each experiment, the adsorbent was pre-treated in-situ in vacuum at a constant temperature 423 K / 3 h for carbon adsorbents and 523 K / 6 h

for zeolites.

The working of the adsorption device for separating the $^{16}O_2$ and $^{18}O_2$ is as follows:

1) The pre-weighted amount of microporous adsorbent is filled in sample cell and pretreated in vacuum at 423 K / 3 h for carbon adsorbents and 523 K / 6 h for zeolites to remove adsorbed moisture and other gases.

2) Before introducing the isotope gases, valves V1-V10 are kept open and evacuated using the vacuum pumps P1, P2 and P3.

3) Close the valves once vacuum is attained.

4) Fixed volume of isotope gases are introduced from T1 and T2 through C and allowed to diffuse overnight to form uniformly mixture and stores in reservoir S1.

5) Close valve V1 and V3 and open other valves to evacuate the inside pipes of the system.

6) Close valves V2, V4, V5, V8 and V9. Open V3, V6, V7 and V10 to flow a fixed volume of isotope gas mixture to be analyzed by MS to determine the mole fractional content of individual gas components in the mixture gas.

7) Then open valves to evacuate the system again and close them after complete evacuation. At this stage, open valve V3, V4, V5, V6 and V7 to flow a fixed volume of isotope mixture gas through the adsorbent filled sample cell maintained at 112 K. The isotope mixture gas is evacuated by suction pump P3. On passing through the adsorbent kept at low temperature, $^{18}O_2$ isotope are preferentially adsorbed in the pores of microporous adsorbent. Unadsorbed

isotope mixture gas pumped by P3 is monitored to determine the molar concentration. The molar concentration of oxygen isotope gas mixture is monitored at every adsorption desorption cycle by the MS. The excess leftover mixture gas can be stored in reservoir S2.

8) After complete adsorption, close valves V4 and V5, while other valves are opened for evacuation. Now, the temperature of the adsorbent cell is raised by closing the cryostat. At elevated temperature, adsorbed isotope gas mixture by the adsorbent are desorbed (released).

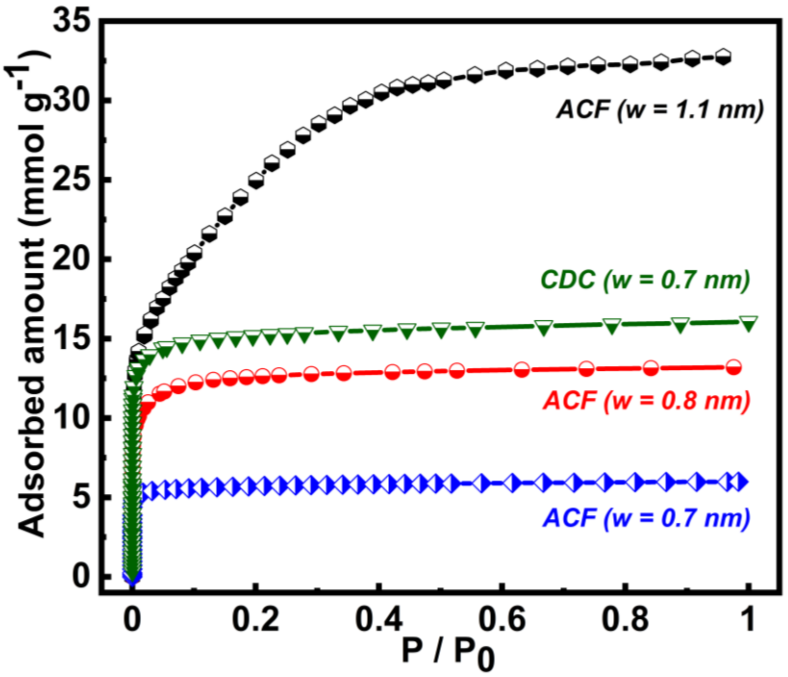


**Supplementary Figure 4. Adsorption Isotherms.** $N_2$ adsorption isotherms of ACF20 (*w* = 1.1 nm), ACF10 (*w* = 0.8 nm), ACF5 (*w* = 0.7 nm), and CDC (*w* = 0.7 nm) at 77 K.

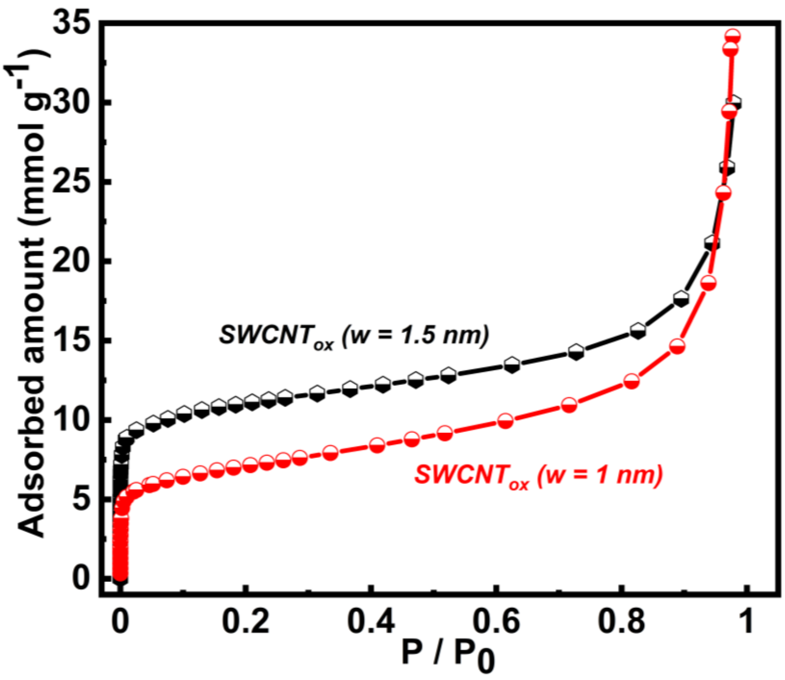


**Supplementary Figure 5. Adsorption Isotherms.** $N_2$ adsorption isotherms of $SWCNT_{ox}$(*w* = 1 nm) and $SWCNT_{ox}$ (*w* = 1.5 nm) at 77 K.

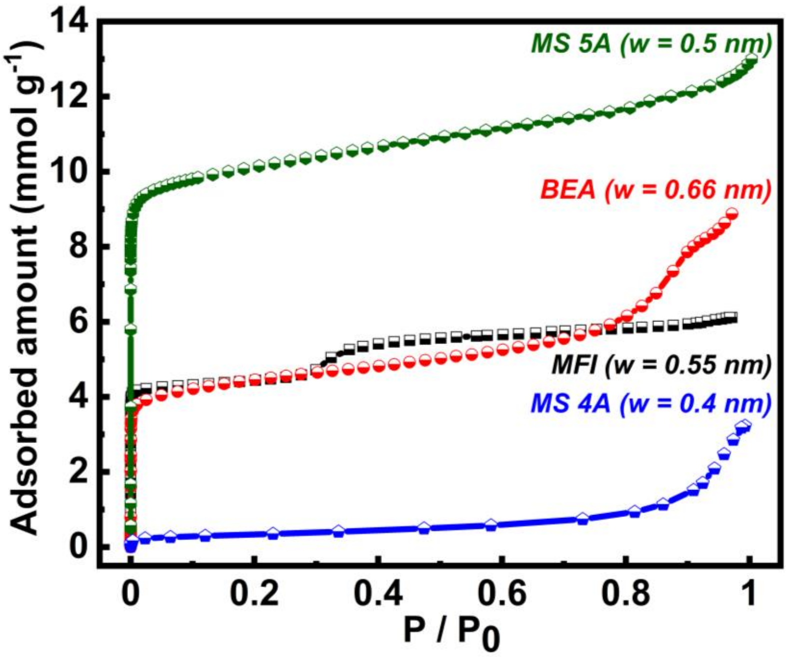


**Supplementary Figure 6. Adsorption Isotherms.** $N_2$ adsorption isotherms of pure silica beta zeolite BEA (*w* = 0.66 nm), silicalite-1 zeolite MFI (*w* = 0.55 nm), MS5A and MS4A at 77 K.

**Supplementary Table 1.** Pore structural parameters determined by $N_2$ adsorption isotherm at 77 K.

The pore width is the effective pore width determined from $N_2$ adsorption isotherms following method discussed in our previous report[2].

| Adsorbent | Specific surface area (BET) ($m^2$ $g^{-1}$) | Specific surface area ($\alpha_s$-analysis) ($m^2$ $g^{-1}$) | Pore volume (ml $g^{-1}$) | Pore width (*w*) (nm) |
|---|---|---|---|---|
| **ACF20** | 1660 | 1520 | 0.81 | 1.1 |
| **ACF10** | 760 | 990 | 0.38 | 0.8 |
| **ACF5** | 520 | 830 | 0.27 | 0.7 |

| | | | | |
|---|---|---|---|---|
| **CDC** | 1340 | 1565 | 0.54 | 0.7 |
| **$SWCNT_{ox}$ (1nm)** | 570 | 645 | 0.21 | 1 |
| **$SWCNT_{ox}$ (1.5 nm)** | 910 | 1150 | 0.36 | 1.5 |
| **BEA** | 375 | 406 | 0.17 | 0.66 |
| **MFI** | 400 | 440 | 0.16 | 0.55 |
| **MS5A** | 560 | 710 | 0.21 | 0.5[a] |
| **MS4A** | 22 | 28 | -- | 0.4[a] |

[a]Pore width values of zeolites are cited from literature[3, 4].

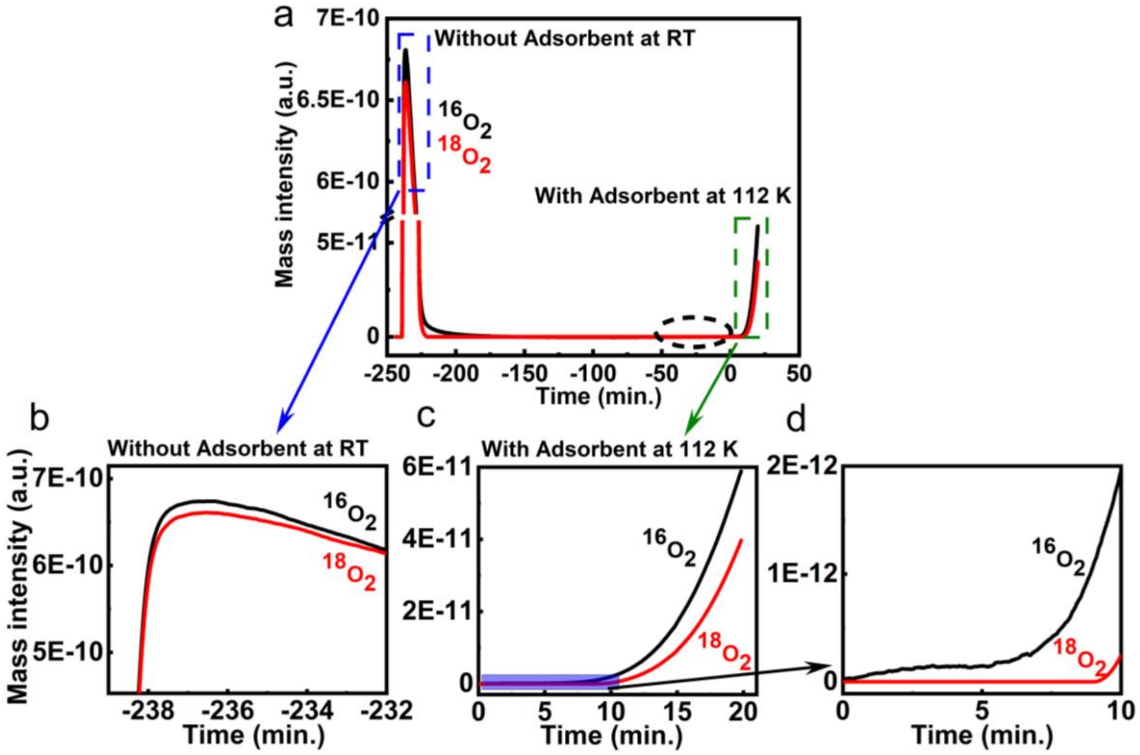


**Supplementary Figure 7. Time course of mass intensity. a,** Time course of mass intensity in two regions: Without adsorbent at room temperature (Blue dashed box) and through adsorbent maintained at 112 K (Green dashed box) for selectivity $S(^{18}O_2/^{16}O_2)$ of CDC at 112 K for the 300 Pa feed gas mixture ($^{18}O_2$ : $^{16}O_2$ = 1 : 1) under dosing rate (~ 6 mL min$^{-1}$). **b, c, & d,** Magnified view.

## Supplementary Note 4:

The MS perform on-line analysis of the unadsorbed mixed gas in gas phase. When mixed gas is initially introduced to the adsorption cell maintained at 112 K, almost all $^{18}O_2$ is adsorbed due to the large pore volume of CDC (0.54 ml g$^{-1}$). In contrast, $^{16}O_2$ molecules, which are more weakly adsorbed, can be detected after a very short time (Supplementary Figure 7**d**) by MS

(almost immediate breakthrough). This can be reflected from the difference in adsorption amount of $^{18}O_2$ and $^{16}O_2$ even in the initial adsorption time. The adsorption selectivity $S$ for $^{18}O_2$ against $^{16}O_2$ is defined as:

$$S(^{18}O_2/^{16}O_2)_{(\mathrm{ads-g})} = \frac{(^{18}O_2/^{16}O_2)_{\mathrm{ads}}}{(^{18}O_2/^{16}O_2)_{\mathrm{g}}}$$

As the dividing factor $(^{18}O_2/^{16}O_2)_g$ is zero, the whole expression becomes infinity. Consequently, the selectivity determination is possible only after $^{18}O_2$ breakthrough the sorbent and can be analyzed by the MS. That is why, even though we can determine the adsorption amount from 0 min. of the adsorption experiment, but selectivity can be analyzed only when the strong sites of pores of CDC are sufficiently filled to allow $^{18}O_2$ molecules to reach MS. The selective adsorption capacity plays extremely significant role in the time period after which selectivity analysis can be performed. This can be well explained using Supplementary Figure 7.

The Supplementary Figure 7**a** shows time course of mass intensity in two regions: Without adsorbent at room temperature (Blue dashed box) and through adsorbent maintained at 112 K (Green dashed box). Supplementary Figure 7**b**, **c** and **d** show their magnified view. The time at which mixed isotope gas is introduced to the adsorbent maintained at 112 K is referred as the initial time (0 min abscissa), hence conventionally, the time without adsorbent region is having negative values. The mole fraction of mixed isotope gas as determined from the no adsorbent region is, $n$ ($^{18}O_2$ : $^{16}O_2$) = 1 (Supplementary Figure 7**b**). The small difference in the mass intensity in no adsorbent region at RT can be related to correlation of mass intensity versus

partial pressure for $^{16}O_2$ and $^{18}O_2$ shown in Supplementary Figure 3 e & f, which exhibits difference in their intensities at particular partial pressure. When the mixed isotope gas is passed through adsorbent maintained at 112 K (Supplementary Figure 7**c** & **d**), almost all isotope gas $^{18}O_2$ is adsorbed for initial 9.7 minutes and the $^{16}O_2$ which remain unadsorbed can only be detected by the MS as can be seen from Supplementary Figure 7**d**. Consequently, the selectivity determination before 9.7 minutes or immediately after exposure to the adsorbent to the mixture in present experiment is not possible. The selectivity can only be determined after 9.7 minutes, when MS starts detecting the $^{18}O_2$ gas molecules also. At 10 minutes, it can be observed that the difference in the mass intensity of $^{18}O_2$ and $^{16}O_2$ is very large, giving high transient selectivity. The above experiment is performed using 300 Pa of mixed oxygen isotope gas introduced at a dosing rate ~ 6 ml $min^{-1}$, so the initial selectivity time is 9.7 min. However, for 120 Pa feed gas introduced at a dosing rate ~ 1 ml $min^{-1}$, the initial time is higher. So, it can be inferred that the initiation of selectivity time is dependent on the amount, dosing rate and molar ratio of feed mixed gas as well as the adsorption temperature.

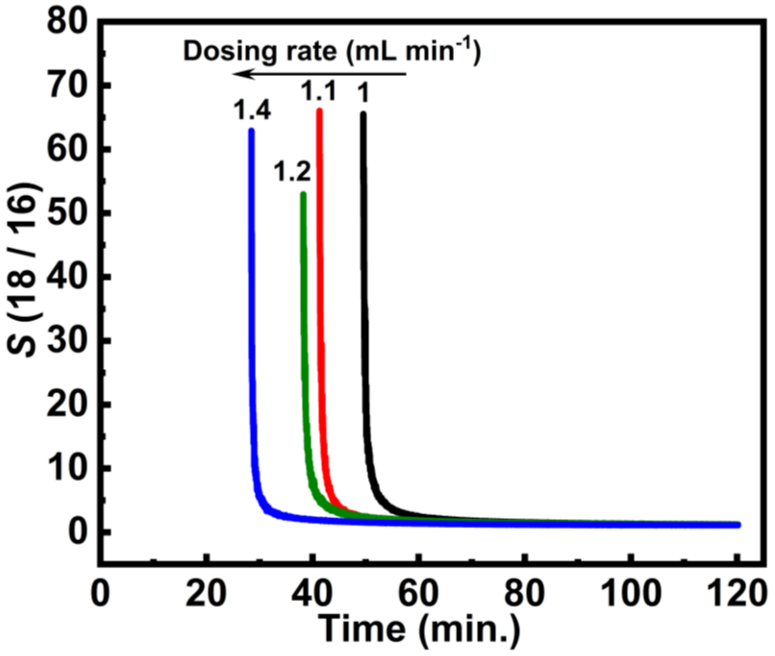


**Supplementary Figure 8. Dosing Rate Dependent CDC Selectivity. a,** Time course of the selectivity *S*($^{18}O_2$/$^{16}O_2$) of CDC at 112 K for 50 % of $^{18}O_2$ in the feed gas mixture at different dosing rates.

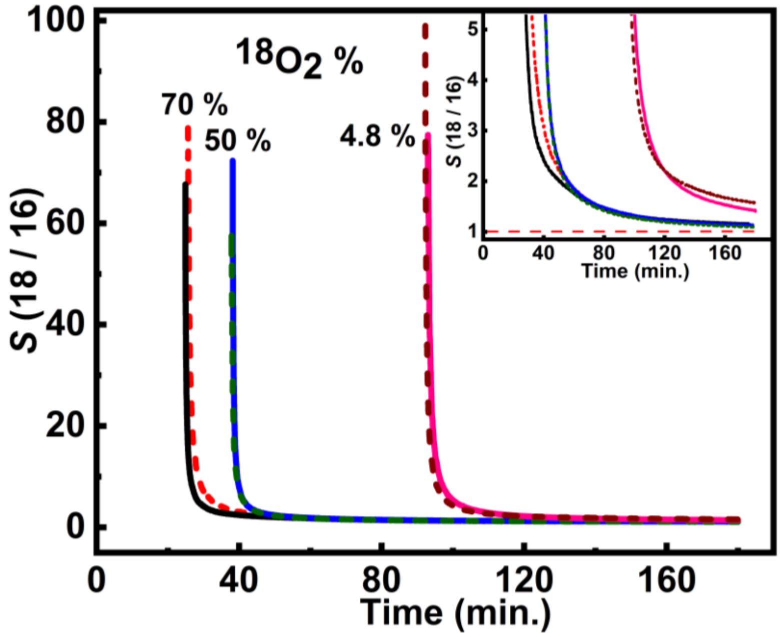


**Supplementary Figure 9. Mole Ratio Dependent CDC Selectivity.** Reproducibility of the selectivity *S*($^{18}O_2$/$^{16}O_2$) of CDC at 112 K for 4.8, 50 and 70 at. % of $^{18}O_2$ in the feed gas mixture under similar dosing rate (~ 1 mL min$^{-1}$). Inset showing the enlarged view. Dotted curves show repeated data.

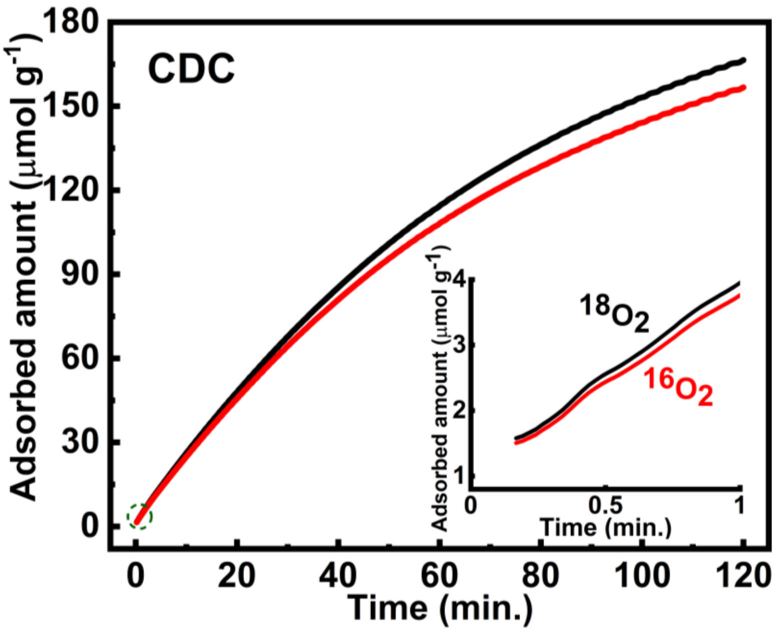


**Supplementary Figure 10. CDC Adsorption Amount.** Time courses of adsorption amount of $^{18}O_2$ and $^{16}O_2$ on CDC at 112 K from equimolar mixed gas. Inset shows magnified view of initial few min.

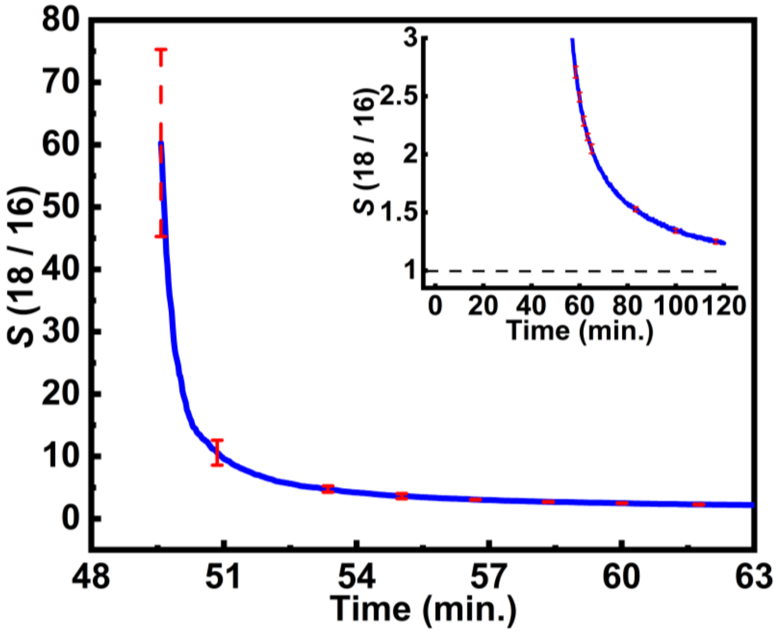


**Supplementary Figure 11. CDC Selectivity at 112 K.** Time courses of $S(^{18}O_2/^{16}O_2)$ on CDC during initial few minutes. Insets demonstrating the $S(^{18}O_2/^{16}O_2)$ for 120 min. Error bars at different time are expressed by red dashed (----) line are standard deviations derived from four measurements.

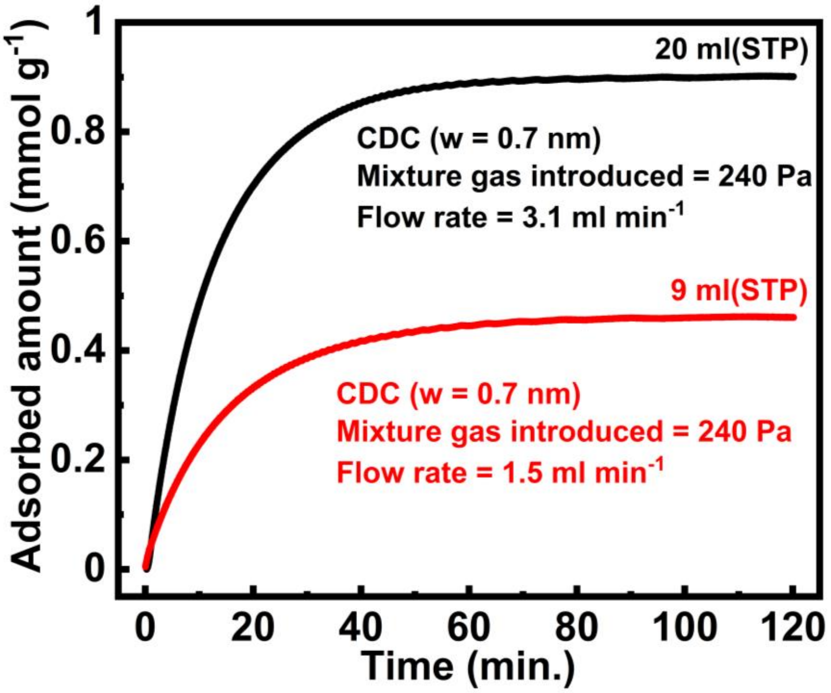


**Supplementary Figure 12. CDC Pore Filling.** Pore volume filling on introduction of $^{18}O_2$-$^{16}O_2$ mixed gas of 240 Pa to CDC at different dosing rate.

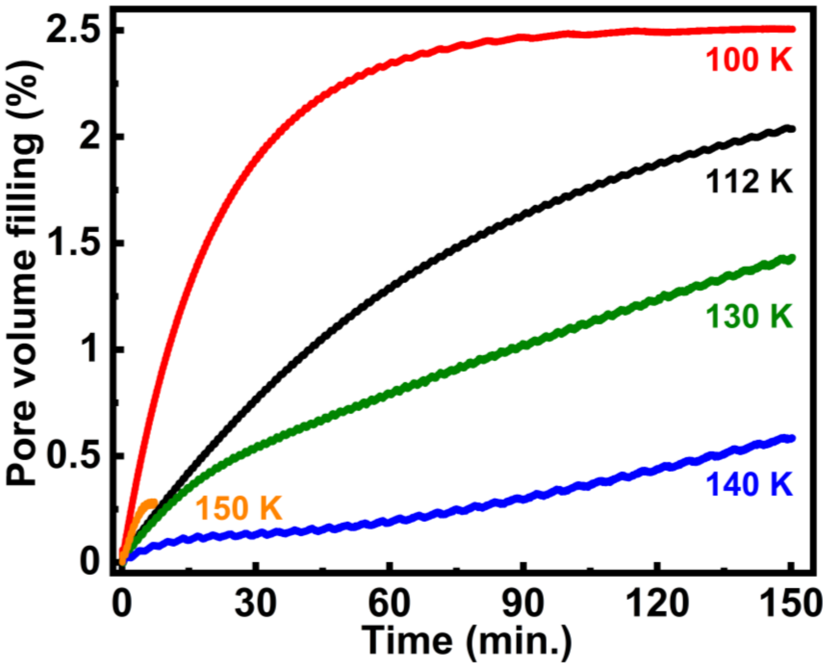


**Supplementary Figure 13. Temperature Dependence of CDC Pore Filling.** Pore volume filling on introduction of $^{18}O_2$-$^{16}O_2$ mixed gas to CDC at different temperatures.

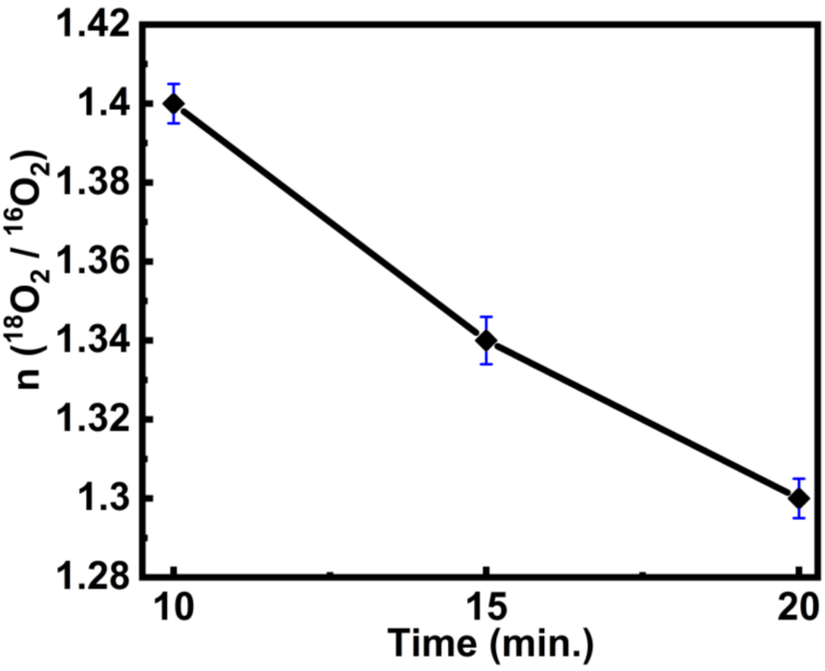


**Supplementary Figure 14. Mole Fraction of Adsorbed Isotope Mixture.** Adsorption amount ratio in mole determined by the amount of desorbed mixed gas, corresponding to 10, 15 and 20 min after adsorption experiment at 112 K. Error bar (blue lines) represent standard deviations derived from four measurements.

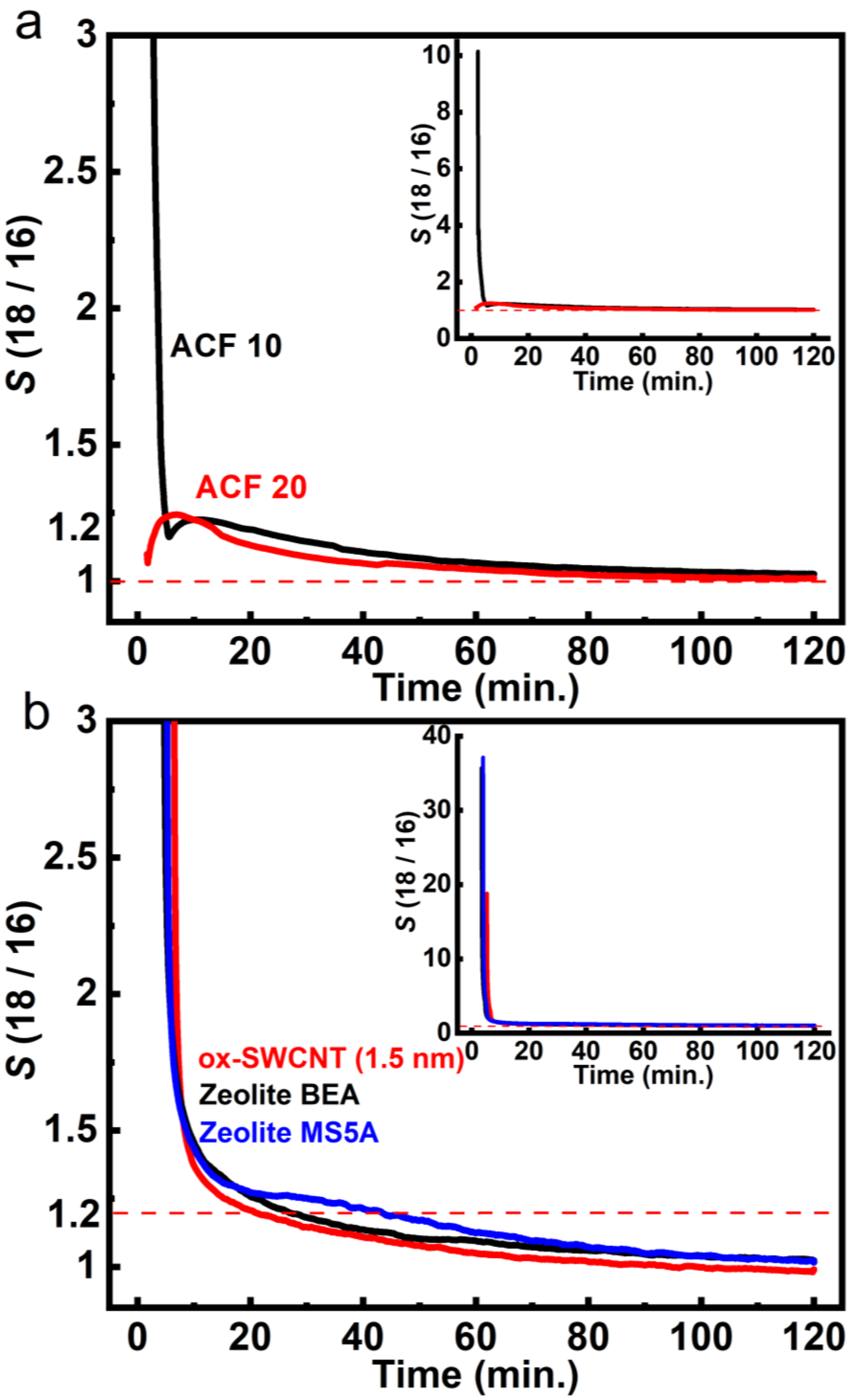


**Supplementary Figure 15. Selectivity of Different Adsorbents.** Time courses of $S(^{18}O_2/^{16}O_2)$ on, **a**, ACF10, ACF20 and **b**, $SWCNT_{ox}$ (1.5 nm), zeolites MS5A and BEA during 120 min. Insets show the time courses with the initial stage.

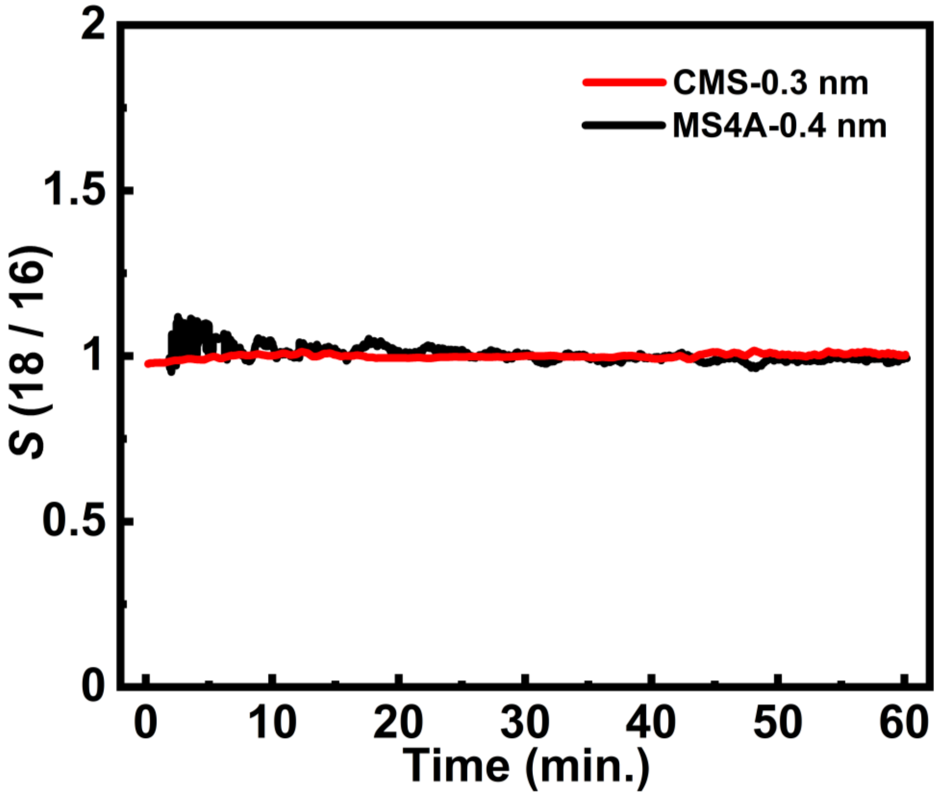


**Supplementary Figure 16. Selectivity of Different Adsorbents.** Time courses of $S(^{18}O_2/^{16}O_2)$ of carbon molecular sieve (CMS) having pore width 0.3 nm and MS4A with pore width 0.4 nm.

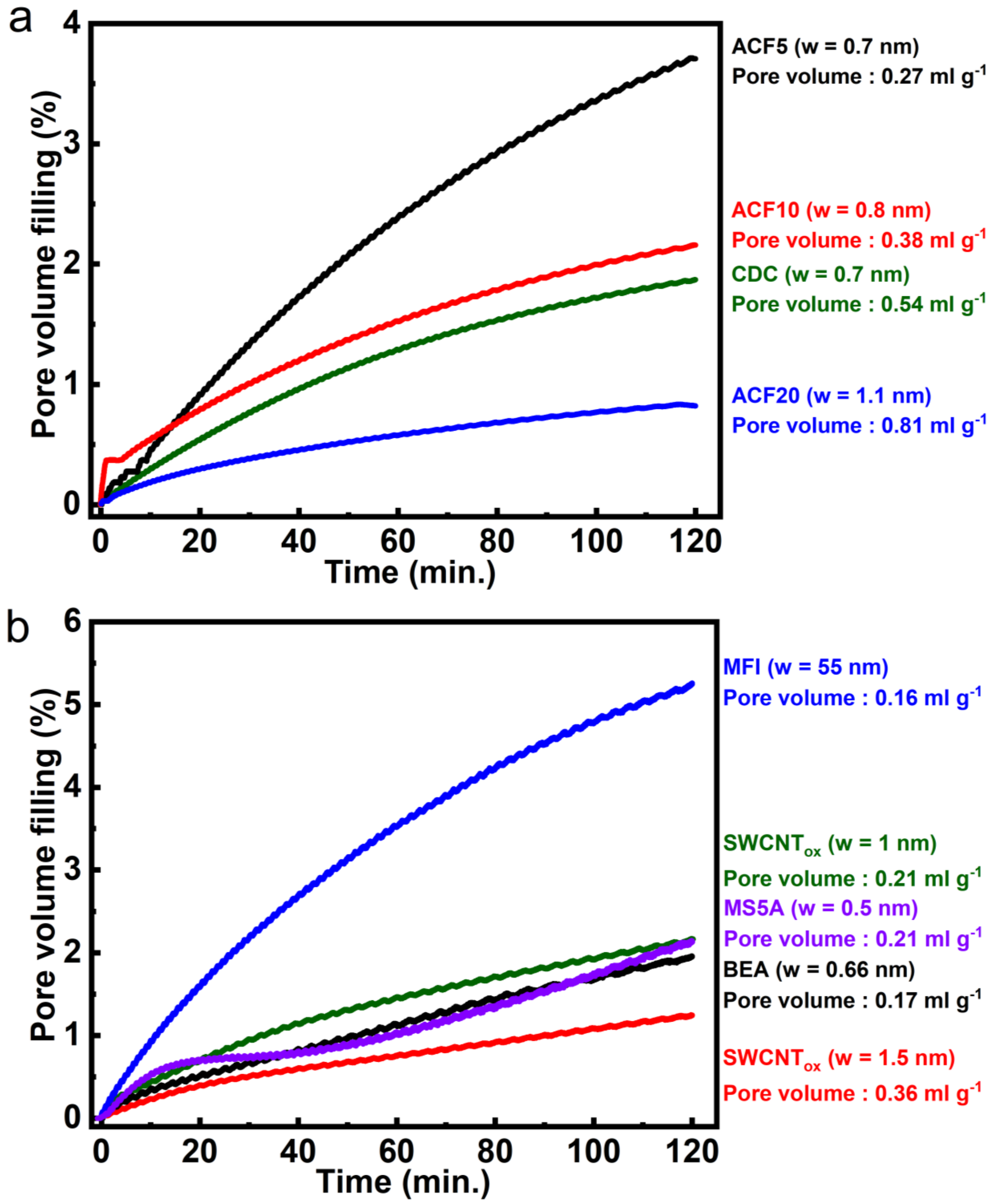


**Supplementary Figure 17. Pore Filling of Different Adsorbents. a, b,** Pore volume filling of $^{18}O_2$-$^{16}O_2$ mixed gas on different nanoporous materials at 112 K.

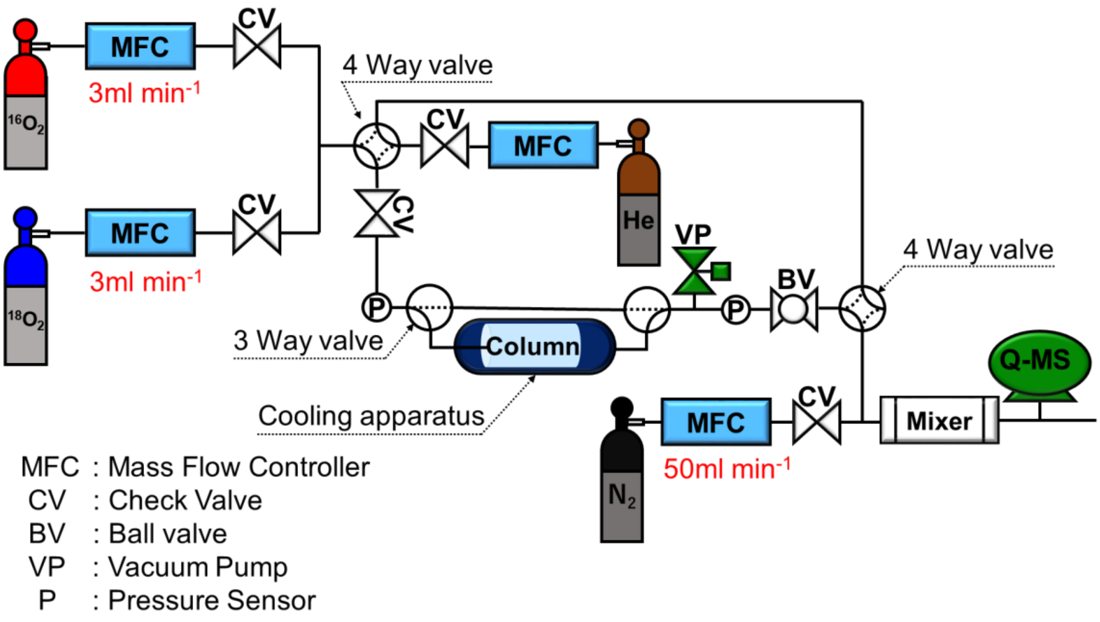


**Supplementary Figure 18. Breakthrough Instrumentation.** The schematic illustrates the custom designed breakthrough measurement instrument.

**Supplementary Note 5:**

Proof-of-concept system for oxygen isotopes separation by low-temperature adsorption. The schematic illustrates the lab-built breakthrough measurement set-up equipped with a mass flow controller, an adsorbent packed column (5 mm diameter and 50-80 mm length) and a quadrupole mass spectrometer (MS). The cryogenic conditions are attained using liquid $N_2$, a heater and a temperature controller. The column is filled with He at 103 K and equimolar mixed gas ($^{16}O_2$ + $^{18}O_2$) is flown through it at a flow rate 6 ml min$^{-1}$. The real-time monitoring of the effluent gas flow through adsorbent column is analyzed to gather information about the separation performance, adsorption kinetic, and selectivity.

**Supplementary Table 2.** The adsorbents, experimental conditions and the selectivity for breakthrough experiment.

| Adsorbent | Weight (g) | Flow rate $^{18}O_2$+$^{16}O_2$ (ml min$^{-1}$) | Concentration $^{18}O_2$+$^{16}O_2$ (%) | Ads. Temp. (K) | Selectivity |
|---|---|---|---|---|---|
| **ACF5** | 0.847 | 6.00 | $^{18}O_2$(50), $^{16}O_2$ (50) | 103 | 1.5 |
| **ACF10** | 0.377 | 6.00 | $^{18}O_2$(50), $^{16}O_2$ (50) | 103 | 3 |

**Supplementary Methods 1:**

**Computational Methodology**

**Equilibrium isotope selectivity**: Isotopic adsorption of $^{16}O_2$ and $^{18}O_2$ in $SWCNT_{ox}$ and silicalite-1 zeolite (MFI) was studied using equilibrium isotope fractionation ratio under two different thermodynamic phases. An equilibrium isotopic exchange reaction between the bulk oxygen gas phase (g) and the nano-confined adsorbed (ads) phase was considered for $SWCNT_{ox}$ and MFI involving two different isotopic species given by the equilibrium expression

$$^{16}O_2(\mathrm{ads}) + {}^{18}O_2(\mathrm{g}) \leftrightharpoons {}^{16}O_2(\mathrm{g}) + {}^{18}O_2(\mathrm{ads}) \qquad \text{(S1)}$$

The equilibrium isotopic separation or selectivity factor computed from simulations is denoted as $\alpha_{(ads\text{-}g)}$ and can be related to the isotopic ratio of $^{18}O_2$ to $^{16}O_2$ within each phase given be the following expression, which is also associated with the change in equilibrium free energy ($\Delta A_{(\mathrm{ads-g})}$),

$$\alpha_{(\mathrm{ads-g})} = \frac{(^{18}\mathrm{O}_2/^{16}\mathrm{O}_2)_{\mathrm{ads}}}{(^{18}\mathrm{O}_2/^{16}\mathrm{O}_2)_{\mathrm{g}}} = e^{-\beta\Delta A_{(\mathrm{ads-g})}} \tag{S2}$$

**Path integral molecular dynamics (PIMD)**: All the PIMD simulations were performed using the universal force engine i-PI[5, 6] for the propagation of nuclear degrees of freedom augmented with the energy and force evaluations performed using the LAMMPS simulation package[7]. These PIMD simulations involved a mapping of the quantum partition function of a system to that of a classical ring polymer Hamiltonian composed of P (= 32) replicas for each atom to sufficiently converge their nuclear quantum kinetic energy. The NVT ensemble was enforced by PILE-G thermostat[8] with a time constant of 10 fs. The isotope fractionation ratio or the selectivity factor was determined from the method of direct scaled-coordinates estimator for isotopic fractionation ratio[9]. To be brief, the protocol involves performing two independent simulations representing two different phases (bulk gas and adsorbed phase) having only naturally abundant isotope (i.e., $^{16}O_2$). For each phase, the PIMD simulation involves the determination of effective partition function undergoing the mass transformation of $^{16}O_2$ to $^{18}O_2$. The ratio of the value of partition functions from both these phases would

result in an efficient and direct computation of selectivity factor. To compute isotopic selectivity, only one randomly chosen $^{16}O_2$ molecule in both the bulk oxygen gas phase and adsorbed phase was chose to perform isotopic mass transformation into $^{18}O_2$ to reflect the dilution conditions of $^{18}O_2$ with in $^{16}O_2$. We have also enhanced the random sampling for the selection of an $^{16}O_2$ molecule participating in the isotopic transformation using several quantum alchemical exchanges[10] per PIMD step that resulted in a better statistical efficiency. All the simulations were performed with a time step of 0.25 ps. We note that these calculations only give equilibrium selectivity, not the transient kinetic selectivity observed in the experiments at short times. However, these calculations can be compared with the experimental selectivities at long times.

**Silicate-1 zeolite MFI:** The initial configurations for the loading of oxygen molecules inside a computational cell of MFI (*a*=40.0 Å, *b*=40.0 Å, *c*=26.76 Å) were generated by performing grand canonical Monte Carlo simulations using the RASPA[11] simulation package at the temperatures of 90.2, 112 and 130 K for a relative pressure loading $P/P_0 = 0.12$. The DREIDING force field[12] was used to model the interactions of zeolite with parameters summarized from He et al.[13]. Adsorption molecular $O_2$ was modelled using a potential described by anharmonic bonding terms[14]. A schematic of the MFI framework with adsorbed oxygen molecules was shown in Supplementary Figure 21.

Our PIMD simulations conducted for the temperatures (90.2, 112 and 130 K)

indicate that the MFI is selective for $^{18}O_2$ over $^{16}O_2$ as shown in Fig. 5b. The amount of adsorbed loadings for each temperature were tabulated in Supplementary Table 4. The value of S (1.09) is almost identical for 90.2 and 112 K with the value slightly decreasing for 130 K (~1.038). Due the complex three-dimensional interconnecting porous network of zeolite MFI with a combination of straight and zigzag channels, the study of equilibrium isotopic selectivity under the zero-loading (one molecule of $O_2$ within the computational domain) conditions could lead to systematic errors. These errors are as a result of insufficient sampling and difficulties arising from disentangling contributions for straight and zigzag channels of porous network using a single $O_2$ molecule adsorbed in the computational domain. However, we have instead used $SWCNT_{ox}$ as the adsorbate candidate for its simplicity of the porous channel to investigate the isotopic selectivity factors associated with the conditions of zero-loading.

**$SWCNT_{ox}$:** To mimic the experimental conditions for $SWCNT_{ox}$, we have performed simulations to compute the equilibrium isotopic selectivity for (7,8) chiral index that corresponds to a diameter of 1.018 nm and length of 5.543 nm. Our PIMD simulations were performed at a temperature of 90.2 K for the configurations equilibrated to the densities at $P/P_0$=0.12 that corresponds to an adsorption loading of 7.37 mmol $g^{-1}$ (experimental loading ~7.0 mmol $g^{-1}$) randomly packed using Packmol[15]. An anharmonic, flexible potential was used to model $SWCNT_{ox}$ adsorbed with $O_2$ molecules[14, 16]. Our reported value of selectivity

for the above simulation conditions amounts to 1.164 for selectivity towards $^{18}O_2$. The simulation protocol involves computing the value of *S* using direct isotopic fractionation ratio estimator[9]. We note here that the simulations were performed only for the periodic and isolated carbon nanotube configurations and any other effects associated with the carbon nanotube bundle configurations or the isotopic selectivities associated with the adsorption of oxygen within interstitial space between the tubes in a bundle were neglected. The randomly packed adsorbed oxygen used as a starting point for the calculations was relaxed using force-field methods, resulting in an ordered packing of $O_2$ molecules (shown in inset Fig. 5a). Such a phenomenon was reported earlier by Maniwa et al.[17] and Sinnott et al.[18] for the formation of different types of ordered packing for the lower temperature structures of oxygen within the nanotubes.

To investigate the dependence of diameter of $SWCNT_{ox}$ towards the isotopic selectivity at the conditions of zero-loading, PIMD simulations were performed for $SWCNT_{ox}$ loaded with just 1 molecule of oxygen. The results are shown in Fig. 5a. For smaller diameter configurations (i.e., for (4,4) and (5,5)), a reasonable selectivity was observed for all the temperatures considered in the study. As the tube diameter increases (i.e., for (7,7) and (10,10)), there was no selectivity observed as seen by the convergence of the value of S towards the value of 1. Comparing these results with the (7,8) SWCNT at high $O_2$ loading, which has a larger

diameter than the (7,7) SWCNT, we see that the selectivity of 1.164 is a result of collective nuclear quantum effects.

**Supplementary Table 3.** Isotope selectivities for the zero-loading of different diameter SWCNT

| Diameter of CNT (nm) | Chiral Index | 90.2 K | 112 K | 130 K |
|---|---|---|---|---|
| 0.5424 | (4,4) | 1.456 ± 0.0024 | 1.327 ± 0.0013 | 1.260 ± 0.0022 |
| 0.6781 | (5,5) | 1.062 ± 0.0022 | 1.043 ± 0.0012 | 1.032 ± 0.0017 |
| 0.95 | (7,7) | 1.004 ± 0.0020 | 1.003 ± 0.0012 | 1.002 ± 0.0011 |
| 1.356 | (10,10) | 1.006 ± 0.0037 | 1.002 ± 0.0014 | 1.005 ± 0.0014 |

**Supplementary Table 4.** Isotope selectivities and simulated loadings for zeolite MFI

| Temperature (K) | $S$ ($^{18}O_2/^{16}O_2$) | Simulated Loading at $P/P_0 = 0.12$ (mmol $g^{-1}$) |
|---|---|---|
| 90.2 | 1.092 ± 0.028 | 5.578 |
| 112 | 1.095 ± 0.019 | 5.466 |
| 130 | 1.038 ± 0.020 | 5.114 |

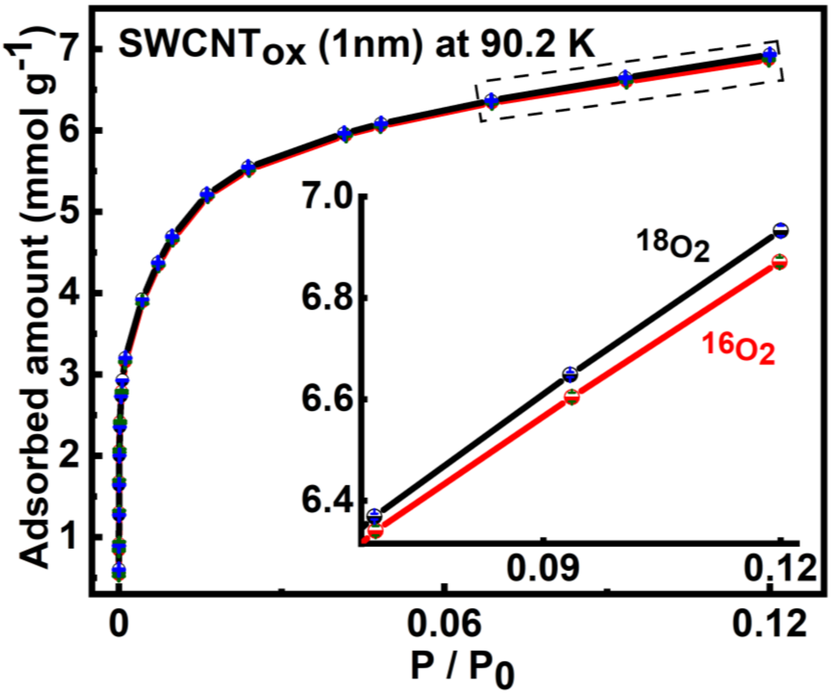


**Supplementary Figure 19. Adsorption Isotherms.** $^{18}O_2$ and $^{16}O_2$ adsorption isotherms of SWCNT$_{ox}$ (1nm) at 90.2 K.

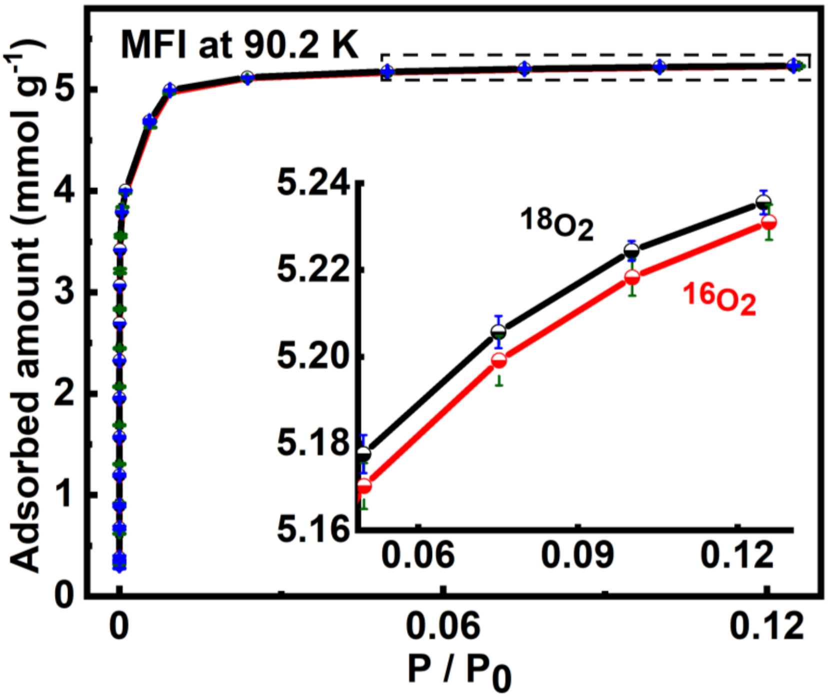


**Supplementary Figure 20. Adsorption Isotherms.** $^{18}O_2$ and $^{16}O_2$ adsorption isotherms of MFI at 90.2 K. Error bars are standard deviations calculated from three measurements.

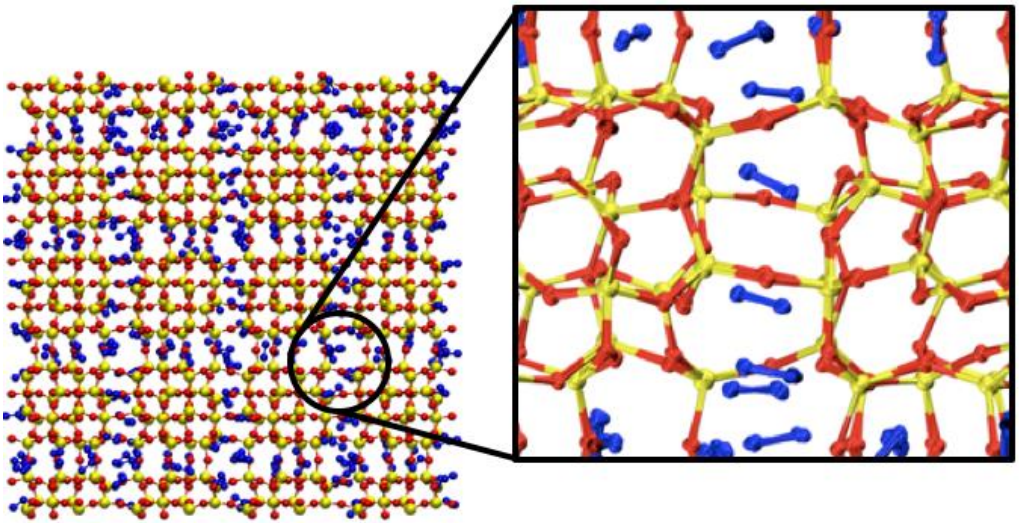

**Supplementary Figure 21. Zeolite MFI structure.** Schematic illustration of Silicate-1 zeolite MFI with adsorbed molecular oxygen corresponding to a loading of 5.578 mmol $g^{-1}$ at a temperature and relative pressure of 90.2 K and $P/P_0$ = 0.12. The enhanced inset shows an illustration of the quantization of 32 replicas per atom within path integrals formalism. Zeolite MFI atoms (silicon in yellow, oxygen in red) and adsorbed oxygen in blue colors.

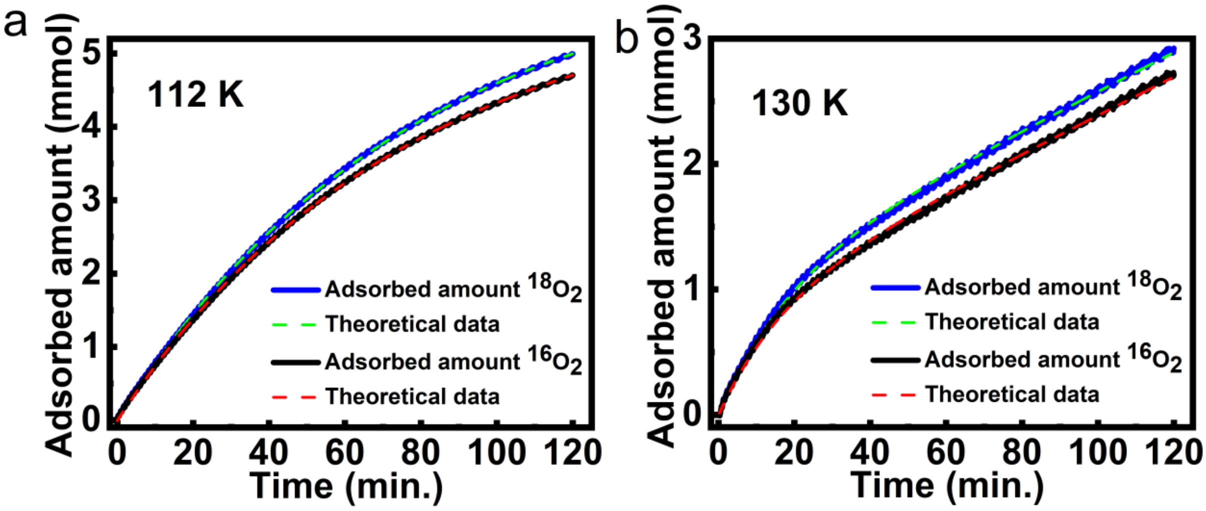


**Supplementary Figure 22. Adsorption Rate of Oxygen Isotopes on CDC.** Adsorption rate of $^{18}O_2$ (blue solid line) and $^{16}O_2$ (black solid line) and theoretical fitting data (dashed line) using linear driving force model at, **a**, 112 K and **b**, 130 K for CDC.

**Supplementary Methods 2:**

**Detailed procedure to determine the rate constant of adsorption and activation energy**

Mathematical simulation of gas separation processes, such as pressure swing adsorption or thermal swing adsorption, (PSA or TSA) requires models for describing adsorption kinetics. The linear driving force (LDF) model, which was originally proposed by Gleuckauf and Coates[19] for adsorption chromatography, is frequently used for this purpose because it is analytical, simple, and physically consistent[20-22].

According to the LDF model, the rate of adsorption of a single adsorbate (pure gas or mixture with an inert gas) into an adsorbent particle is given by:

$$R_{ads} = A[1 - \exp(-kt)] + at \qquad \text{(S3)}$$

*A*: amplitude, *k*: adsorption constant, *t*: time, *a*: fitting parameter of linear function.

**Calculation of $E_a$ using Arrhenius Equation:**

The activation energy can also be calculated directly given two known temperatures and a rate constant at each temperature. Using Equation (S4), suppose that at two different temperatures *T*1 and *T*2, reaction rate constants *k*1 and *k*2:

$$\ln\left(\frac{k1}{k2}\right) = \left(\frac{1}{T2} - \frac{1}{T1}\right)\frac{E_a}{R} \qquad \text{(S4)}$$

The results of adsorption-rate calculations using these equations are reported in Supplementary Figure 22, where a very good agreement with experimental data can be observed.

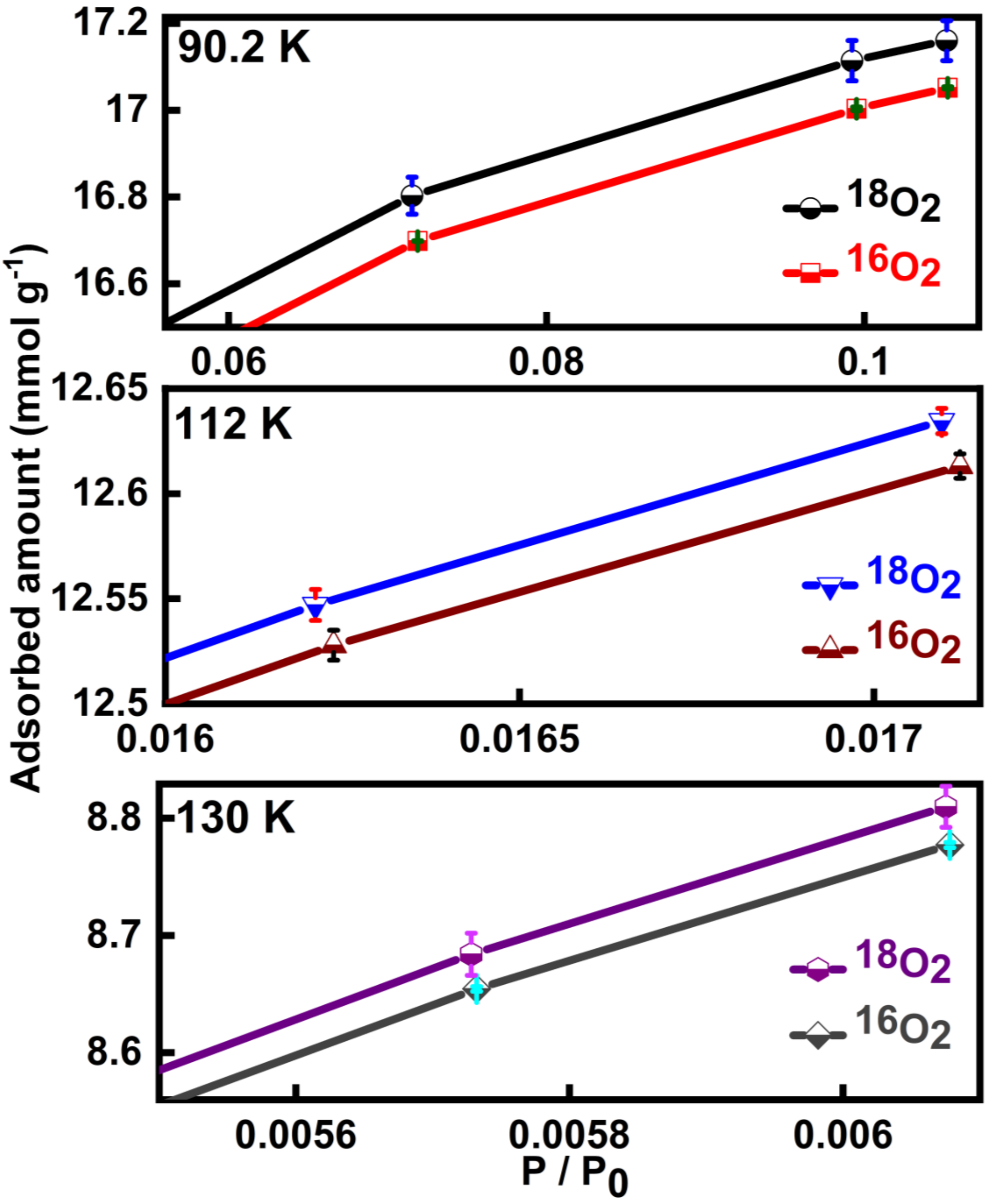


**Supplementary Figure 23. Magnified Adsorption Isotherms.** Enlarged view of $^{18}O_2$ and $^{16}O_2$ adsorption isotherms for CDC at 90.2, 112 and 130 K. Error bars are standard deviations calculated from three measurements.

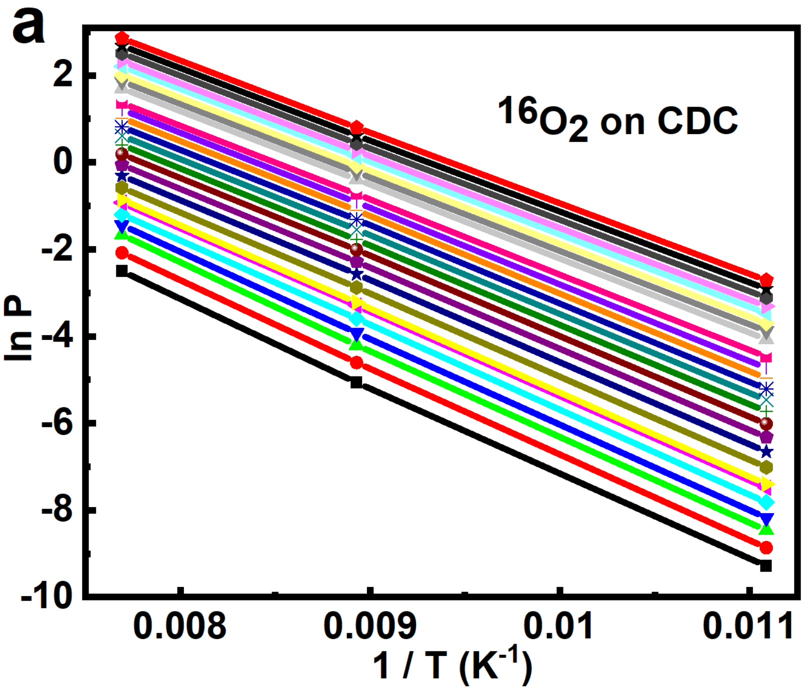


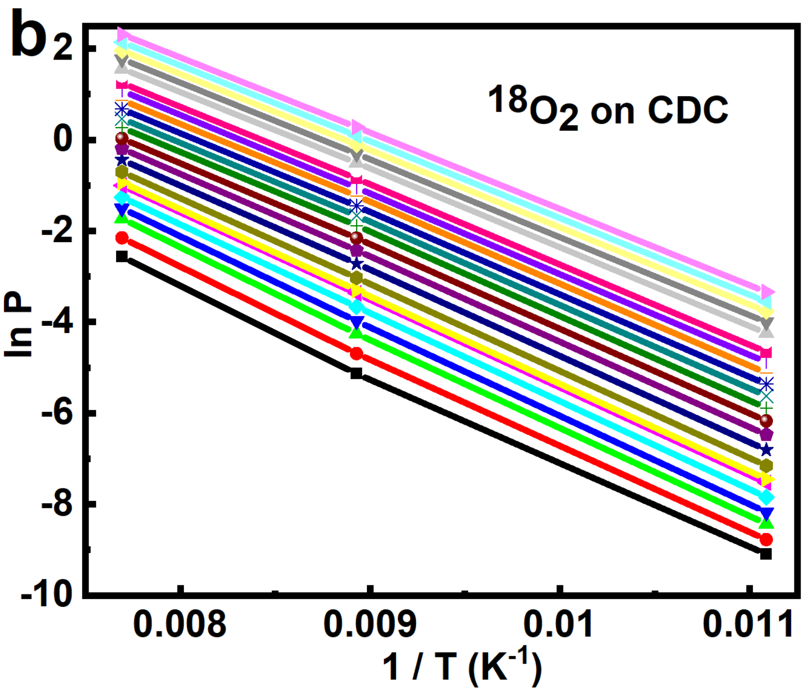


**Supplementary Figure 24. CDC Clausius-Clapeyron plots**. Clausius-Clapeyron plots for adsorption of $^{16}O_2$ and $^{18}O_2$ on CDC for different fractional fillings.

**Supplementary Methods 3:**

**Determination of Isosteric heat of adsorption**

The isosteric heat of adsorption, $q_{st}$, was determined using Clausius-Clapeyron equation for adsorption isotherms of $^{16}O_2$ and $^{18}O_2$ at 90.2 K, 112 K and 130 K. The differential form of the Clausius-Clapeyron equation is given below.

$$\left(\frac{d \ln P}{dT}\right)_{\text{fractional filling}} = \frac{q_{st}}{RT^2} \tag{S5}$$

Using a linear interpolation in the adsorption isotherms for different temperatures, we used the equilibrium pressure ($P$) for a fixed amount of adsorbed gas ($n_a$). We obtained linear relations between the ln($P$) and the reciprocal temperature $1/T$, giving the $q_{st}$ values as a function of the fractional filling.

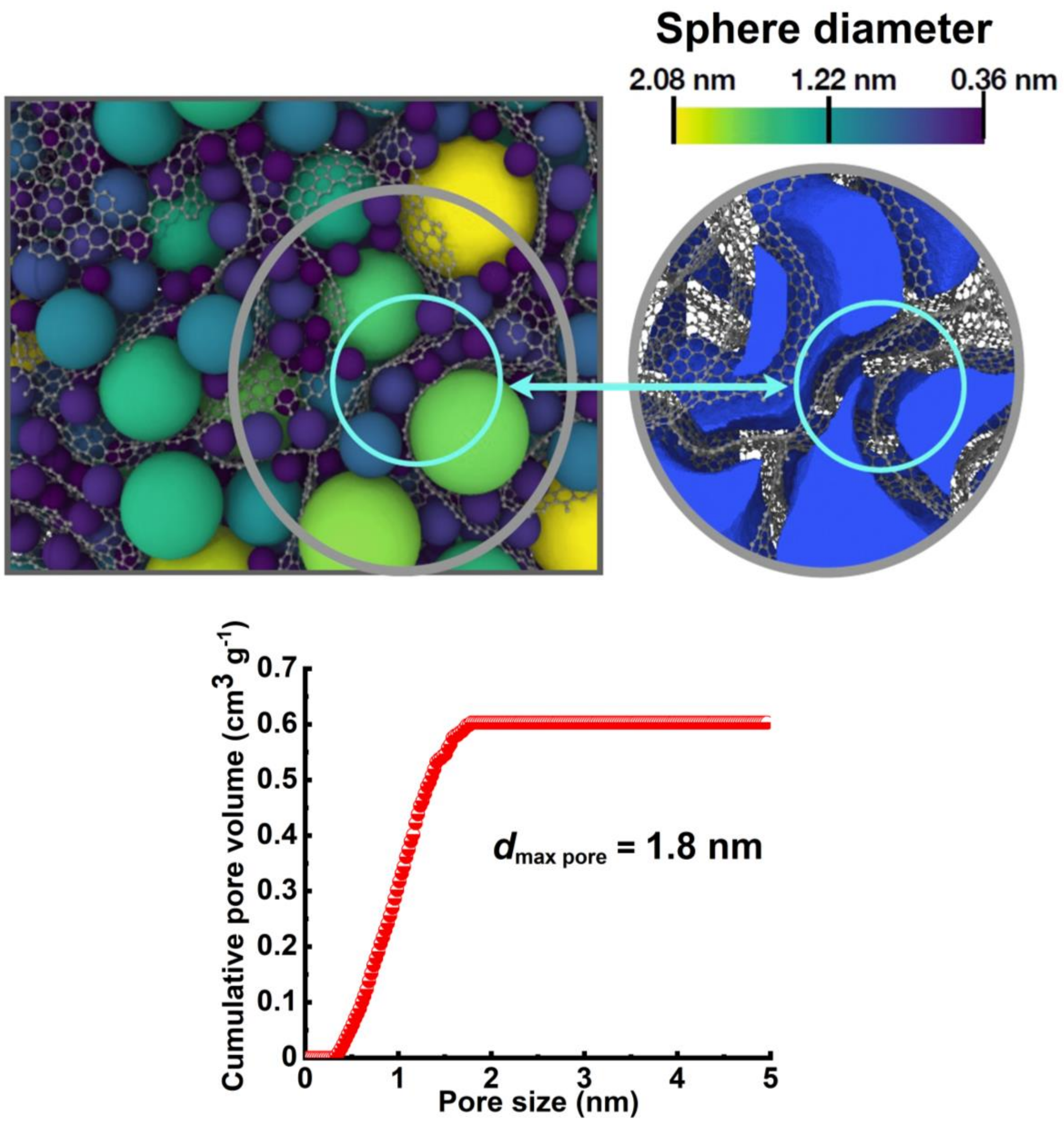


**Supplementary Figure 25. CDC Structure Determination.** Left: detail of the CDC porous structure filled with non-overlapping spheres (Gubbins' method) indicating geometric pore sizes. Cyan circle encloses narrow pore site. Right: Same detail of the CDC structure containing the adsorbed nitrogen network (blue volume). Below: geometric cumulative pore volume from nitrogen adsorption simulation.

**Supplementary References:**


1. Noguchi, D. et al. Quantum Sieving Effect of Three-Dimensional Cu-based Organic Framework for $H_2$ and $D_2$. *J. Am. Chem. Soc.* **130**, 6367–6372 (2008).
2. Kaneko, K., Cracknell, R. F. & Nicholson, D. Nitrogen Adsorption in Slit Pores at Ambient Temperatures: Comparison of Simulation and Experiment. *Langmuir* **10**, 4606–4609 (1994).
3. Herron, N. & Corbin, D. R. (Eds.) in *Inclusion Chemistry with Zeolites: Nanoscale Materials by Design* (Kluwer Academic Publishers: London, 1995) pp 7.
4. Szostak, R. *Handbook of Molecular Sieves* (Van Nostrand Reinhold: New York, 1984) pp. 258.
5. Kapil, V. et al. ipi 2.0: A universal force engine for advanced molecular simulations. *Comput. Phys. Commun.* **236**, 214-223 (2019).
6. Ceriotti, M., More, J. & Manolopoulos, D. E. ipi: A python interface for ab initio path integral molecular dynamics simulations. *Comput. Phys. Commun.* **185**, 1019-1026 (2014).
7. Plimpton, S. Fast parallel algorithms for short range molecular dynamics. *J. Comput. Phys.* **117**, 1-19 (1995).
8. Glesener, J. W., Morrish, A. A. & Snail, K. A. A thinfilm Schottky diode fabricated from flamegrown diamond. *J. Appl. Phys.* **70**, 5144–5146 (1991).
9. Cheng, B. & Ceriotti. M. Direct path integral estimators for isotope fractionation ratios. *J. Chem. Phys.* **141**, 244112 (2014).
10. Liu, J. et al. A surface-specific isotope effect in mixtures of light and heavy water. *J. Phys. Chem. C* **117**, 2944-2951 (2013).
11. Dubbeldam, D., Calero, S., Ellis, D. E. & Snurr, R. Q. RASPA: molecular simulation software for adsorption and diffusion in flexible nanoporous materials. *Mol. Simulat.* **42**, 81–101 (2015).
12. Mayo, S. L., Olafson, B. D. & Goddard, W. A. DREIDING: a generic force field for molecular simulations. *J. Phys. Chem.* **94**, 8897–8909 (1990).
13. Cheng, X., Li, Z. & He, Y.-L. Effects of temperature and pore structure on the release of methane in zeolite nanochannels. *RSC Adv.* **9**, 9546–9554 (2019).
14. Cervellera, V. R., Albertí, M. & Huartelarrañaga, F. A molecular dynamics simulation of air adsorption in single-walled carbon nanotube bundles. *Int. J. Quantum Chem.* **108**, 1714-1720 (2008).
15. Martínez, L., Andrade, R., Birgin, E. G. & Martínez, J. M. Packmol: A package for building initial configurations for molecular dynamics simulations. *J. Comput. Chem.* **30**, 2157-2164 (2009).
16. Alexiadis, A. & Kassinos, S. Molecular simulation of water in carbon nanotubes. *Chem. Rev.* **108**, 5014-5034 (2008).

17. Hanami, K.-I. et al. One-Dimensional Oxygen and Helical Oxygen Nanotubes inside Carbon Nanotubes. *J. Phys. Soc. Jpn.* **79**, 023601 (2010).
18. Lee, K.-H. & Sinnott, S. B. Equilibrium and Nonequilibrium Transport of Oxygen in Carbon Nanotubes. *Nano Lett.* **5**, 793-798 (2005).
19. Gleuckauf, E. & Coates, J. I. The Influence of Incomplete Equilibrium on the Front Boundary of Chromatograms and the Effectiveness of Separation. *J. Chem. Soc.* 1315–1321 (1947).
20. Hartzog, D. G. & Sircar, S. Sensitivity of PSA Process Performance to Input Variables. *Adsorption* **1**, 133 (1995).
21. Gemmingen, *U.V. Pressure Swing Adsorption Process—Design and Simulations.* M. Suzuki (Ed.) (Proceedings of IVth Int. Conf. on Fundamentals of Adsorption, Kyoto, Kodansha: Japan, 1993) pp. 703–712.
22. Chihara, K. & Suzuki, M. Simulation of Non isothermal Pressure Swing Adsorption. *J. Chem. Eng. Jpn.* **16**, 53–61 (1983).
